# Accelerated SPECT image reconstruction with FBP and an image enhancement convolutional neural network

**DOI:** 10.1186/s40658-019-0252-0

**Published:** 2019-07-29

**Authors:** Martijn M. A. Dietze, Woutjan Branderhorst, Britt Kunnen, Max A. Viergever, Hugo W. A. M. de Jong

**Affiliations:** 1 Radiology and Nuclear Medicine, Utrecht University and University Medical Center Utrecht, P.O. Box 85500, 3508 Utrecht, GA Netherlands; 2 Image Sciences Institute, Utrecht University and University Medical Center Utrecht, P.O. Box 85500, 3508 Utrecht, GA Netherlands

**Keywords:** SPECT, Deep learning, Radioembolization, Reconstruction

## Abstract

**Background:**

Monte Carlo-based iterative reconstruction to correct for photon scatter and collimator effects has been proven to be superior over analytical correction schemes in single-photon emission computed tomography (SPECT/CT), but it is currently not commonly used in daily clinical practice due to the long associated reconstruction times. We propose to use a convolutional neural network (CNN) to upgrade fast filtered back projection (FBP) image quality so that reconstructions comparable in quality to the Monte Carlo-based reconstruction can be obtained within seconds.

**Results:**

A total of 128 technetium-99m macroaggregated albumin pre-treatment SPECT/CT scans used to guide hepatic radioembolization were available. Four reconstruction methods were compared: FBP, clinical reconstruction, Monte Carlo-based reconstruction, and the neural network approach. The CNN generated reconstructions in 5 sec, whereas clinical reconstruction took 5 min and the Monte Carlo-based reconstruction took 19 min. The mean squared error of the neural network approach in the validation set was between that of the Monte Carlo-based and clinical reconstruction, and the lung shunting fraction difference was lower than 2 percent point. A phantom experiment showed that quantitative measures required in radioembolization were accurately retrieved from the CNN-generated reconstructions.

**Conclusions:**

FBP with an image enhancement neural network provides SPECT reconstructions with quality close to that obtained with Monte Carlo-based reconstruction within seconds.

## Background

Monte Carlo-based iterative reconstruction to correct for scatter and collimator effects has been proven to be superior over more analytical correction schemes, albeit at the cost of reconstruction times of potentially several hours [1–3]. This has up to now limited introduction of such techniques into daily clinical practice because the requirement of reconstruction job queues causing interrupted workflow is considered bothersome. In addition, technologists prefer to quickly judge whether a scan was correctly performed, e.g., by validating whether a patient has moved during the examination. Substantial reconstruction acceleration can be achieved with code parallelization, the use of dedicated workstations, and restrictions on the matrix size or number of iterations, which might bring the reconstruction time to a clinically acceptable value. We will in this work, however, explore a different method for acceleration that is also less dependent on reconstruction settings for its required reconstruction time.

Deep learning has emerged in recent years for a variety of applications in medical imaging, such as segmentation, super-resolution, and denoising [4]. We believe there is a major opportunity for these networks to aid single-photon emission computed tomography (SPECT) by accelerating the reconstruction step. Specifically, we propose to first generate a low-quality reconstruction using fast filtered back projection (FBP) and then pass the result to an image enhancement convolutional neural network. Such a trained network would improve reconstruction quality and would be able to generate its results within seconds.

Although our approach is valid for all forms of SPECT/CT imaging, the focus of this work will be on potential implementation in the hepatic radioembolization workflow [5]. In this treatment, microspheres filled with radioactivity are inserted into the liver to deliver a damaging dose to the tumour. A scan of technetium-99m macroaggregated albumin (^99m^Tc-MAA) is normally performed in a separate safety procedure for estimation of the lung shunting fraction (LSF) and detection of potential extrahepatic distributions. Ideally, both this pre-treatment procedure and the treatment itself would be performed in a single setting, to minimize in-between changes in anatomy and catheter position [6]. Such a single-session procedure would require SPECT/CT in the intervention room for a smooth workflow, for which we are developing a compact mobile system [7–9], scanning to be performed in minutes, and reconstructions to be available within seconds.

This study compares the obtained image quality, reconstruction speed, and response to short scan time for the discussed fast neural network approach, a relatively slow but accurate Monte Carlo-based reconstructor, and a commercially available clinical reconstructor, so that it becomes clear whether the neural network approach can provide reconstructions with quality similar to those obtained with Monte Carlo-based reconstruction, but now within seconds.

## Methods

Convolutional neural networks are usually trained with pairs of low- and high-quality images. In nuclear medicine, however, the high-quality images (e.g. obtained with long scan time) can be substantially different from true distributions, due to technical limitations such as the limited spatial and energy resolution of the gamma camera. To prevent the neural network from learning the errors that arise from these limitations, this work introduced an additional intermediate reconstruction and projection step. The neural network could with this method potentially learn how to approach the true distribution better than the iterative image reconstructor. The separate steps are discussed in more detail below.

### Patient data

Our retrospective study was approved by the local ethics committee, who also waived the need for informed consent of the patients involved. Projections of 128 SPECT/CT scans from the pre-treatment ^99m^Tc-MAA radioembolization procedure were available. All scans were performed on a dual-head Symbia T16 detector system (Siemens Healthineers, Erlangen, Germany). Projections were obtained in 20 min under 120 angles using a low energy high resolution (LEHR) parallel hole collimator with a photopeak window between 129 and 150 keV and a scatter window between 108 and 129 keV. Out of the 128 distributions, 100 were used for network training, 20 for validation, and 8 for testing purposes.

### Ground truth reconstruction

The patient projections were first reconstructed using the Utrecht Monte Carlo System (UMCS) [10]. This software package has been validated for several isotopes [11–14] and is considered state-of-the-art. UMCS accounts for attenuation with the μ-map obtained from the CT, resolution through point spread function modelling, and scatter using Monte Carlo simulation of the photon interactions in the body. A total of 10 iterations with 8 subsets were performed and no post-reconstruction filter was applied. The volumes had 128 × 128 × 128 voxels with 3.9 mm isotropic voxel size. The resulting reconstructions were set as ground truth distributions and were used for comparison of the reconstruction methods at a later stage.

### Synthetic volumes

The performance of the neural network should improve when more unique volumes are available to train on. The 100 ground truth distributions in the training set were hence used to create additional synthetic volumes. From a random patient, the liver mask with corresponding attenuation map was first selected. A sphere with a random diameter of 7 to 20 pixels was then positioned at a random location in the liver and filled with a random patch of the activity distribution from another patient. The process was repeated until the entire liver mask was filled. The generated synthetic volumes were thus a composition of patches from tens of separate reconstructions. In total, 900 synthetic volumes were created with this method.

### Projection generation

Projections of the 100 ground truth distributions and 900 synthetic volumes (with collimator and detector effects and up to ten orders of scatter) were generated using UMCS. The use of a high number of photon tracks combined with convolution-based forced detection yielded nearly noise-free projections. Poisson noise was then added so that the simulated projections became representative of real detector measurements. Projections were simulated for two scan times: 20 min, as is customary for a regular diagnostic SPECT/CT scan, and 5 min, which we envision for use in interventional radioembolization procedures. The total activity of the ground truth volumes was set to 150 MBq, as this is the average injected dose in the radioembolization pre-treatment procedure in our hospital. The detector was configured with a single head, in anticipation of the compact mobile system mentioned in the introduction.

### Reconstruction methods

The above projection sets were reconstructed using four different methods:

- Filtered back projection (FBP). The ramp filter was used in combination with Chang’s correction [15] to compensate for attenuation (using the attenuation map from the CT scan). A post-reconstruction Gaussian filter of 5 mm full width at half maximum (FWHM) was applied to remove the most severe artefacts.
- Monte Carlo-based reconstruction (MC). The projections were reconstructed using the same reconstructor as used in the initial reconstruction (UMCS). A total of 10 iterations with 8 subsets were performed and no post-reconstruction filter was applied.
- Clinical reconstruction (CLINIC). An iterative reconstruction method, as can be found in state-of-the-art clinical methods (such as Flash3D in Siemens systems), was used. This reconstruction method included attenuation correction and resolution recovery and used dual-energy window scatter correction [16]. Scattered photons were smoothed with a Gaussian filter of 5 mm FWHM and added to the reconstruction loop at fraction *k* = 0.5. A post-reconstruction Gaussian filter of 5 mm FWHM was employed and a total of 10 iterations with 8 subsets were performed. These settings were chosen as they are the current clinical practice in our institute.
- Convolutional neural network approach (CNN). The projections were first reconstructed using FBP as above and then fed to the trained network to increase the image quality.

### Network design

**Fig. 1:**
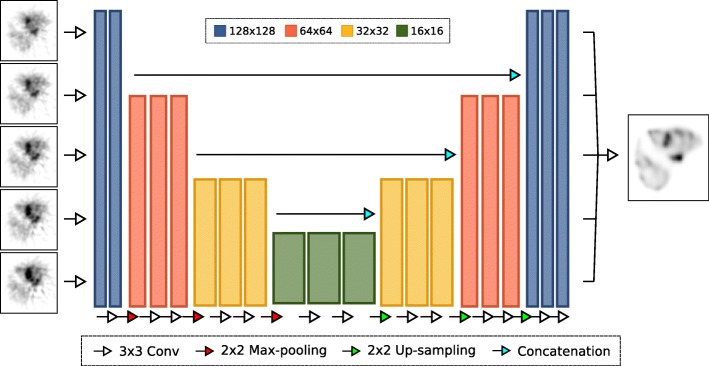
Schematic of the encoder-decoder convolutional neural network with five input slices as used in this study. Left are examples of the FBP input reconstructions and right the associated ground truth distribution

The neural network used a deep convolutional encoder-decoder structure (see Fig. 1), which is frequently used for denoising applications [17, 18]. Network training was performed by minimizing the voxel-wise mean squared error of the FBP reconstructions with the combination of the 100 ground truth distributions and 900 synthetic volumes. The network consisted of layers with several resolutions, which were connected with each other via concatenation (to ensure small objects were not lost in training). Five adjacent slices per sample were used as input so that resolution in all directions was preserved. By inserting all 128 slices from FBP into the network, the entire volume was reconstructed. Separate networks were trained for both simulated scan times.

For the encoding layers, every step consisted of two 3 × 3 convolutional layers with ReLu activation function, followed by 2 × 2 max pooling. The decoding layers first upsampled the resolution and again used two 3 × 3 convolutional layers. Learning was performed using the ADAM optimizer [19] with a learning rate of 1e−4. The data was fed to the network with a batch size of 128. Training continued until no further decrease in the loss function was observed for 20 epochs. The training was performed using TensorFlow 1.7.0 [20] with Keras 2.1.6 [21].

### Evaluation

#### Network performance

It was first studied whether the introduced reconstruction and projection step (by setting the initial reconstructed images as ground truth) performed better than when training directly to the Monte Carlo-based generated reconstructions. It was subsequently evaluated whether the augmentation of training data with synthetic volumes aided network performance by separately training with 0, 300, 600, and 900 synthetic phantoms, in addition to the 100 ground truth distributions. The minimum acquired losses were used as a measure for network performance. Since the neural network is slightly sensitive to the random initial weights chosen, five realizations were performed per setting.

#### Validation performance

The mean squared error of the four reconstructions (normalized to the total reconstructed activity) with the associated ground truth was calculated for the two scan times and used as a quantitative measure for reconstruction quality for the 20 patients in the validation set. The difference of the LSF with the ground truth distributions was furthermore measured because this measure is often assessed in hepatic radioembolization.

#### Phantom measurements

**Fig. 2:**
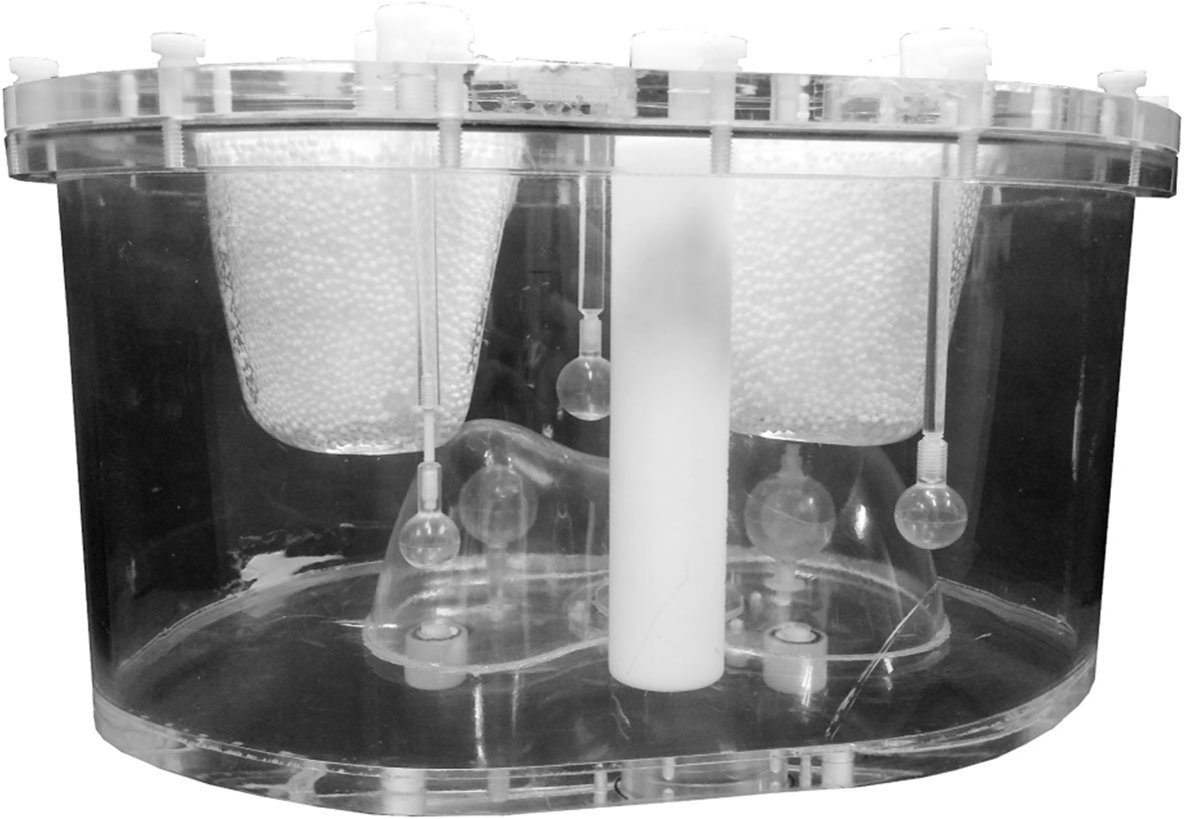
The anthropomorphic phantom with three extrahepatic volumes and one solid lesion and one lesion with a cold core inside the liver

A phantom study was performed to evaluate to what extent the neural network approach could reconstruct true detector projections. An anthropomorphic phantom was adjusted from a commercially available phantom (Anthropomorphic Torso Phantom: ECT/TOR/P) by the inclusion of three extrahepatic spheres (with volumes of 2.0, 4.1, and 8.1 mL) and one solid sphere (15.7 mL) and one sphere with cold core (5.6 mL cold volume; 18.7 mL hot volume) inside the liver (see Fig. 2). The extrahepatic spheres were filled with 2.7 uptake ratio in relation to the liver background activity, for the spheres inside the liver, this ratio was 7.7. The lungs were filled with LSF of 5.2%. The phantom was filled with water and had 157 MBq total activity of ^99m^Tc. The phantom was configured in this way to represent situations encountered in hepatic radioembolization [22, 23].

The anthropomorphic phantom was scanned for 20 min on the same scanner with the same acquisition settings as in the patient scans but now with a single head. By uniform random subsampling from the obtained projections, projections with 5 min scan time were additionally acquired. The liver, sphere, and lung masks were generated from delineation on the low-dose CT. The uptake ratios of the solid spheres, contrast-to-noise ratio (CNR), and LSF were calculated from the reconstructions and compared with the values of the phantom. The CNR was calculated with the solid sphere inside the liver and the liver background.

#### Original detector projections

Reconstructions were finally performed on the 8 patient projections in the testing set to give an indication of the reconstruction performance on true detector projections for patient distributions with varying levels of activity. Since no ground truth is present for these cases, the reconstructions were solely visually compared.

## Results

### Reconstruction time

The CNN reconstruction was performed on the graphics processing unit (GPU), the other options used the central processing unit (CPU) with a single core, all on a Dell Precision 7810 (2.60 GHz Xeon E5-2640v3 and 64 GB RAM) with NVIDIA Quadro P6000. The time for reconstruction of an entire volume was approximately 2 s using FBP and 5 s with CNN. The clinical reconstruction required 5 min and the Monte Carlo-based reconstruction required 19 min. The CNN training took approximately 10 h for the case of 900 synthetic volumes.

### Network performance

Directly training the FBP to the Monte Carlo-based reconstructions (with no synthetic volumes) resulted in a minimum validation loss of 1.31 ± 0.02, whereas with the introduced reconstruction and projection step, it reduced to 1.22 ± 0.01. This indicated that the introduction of this step aided network performance.

The minimum validation loss was 1.22 ± 0.01 when no synthetic volumes were used and then decreased to 1.06 ± 0.02 for 300 volumes, 1.04 ± 0.01 for 600 volumes, and 1.01 ± 0.01 for 900 volumes. The introduction of synthetic volumes into the training set thus also improved network performance.

### Validation performance

**Fig. 3:**
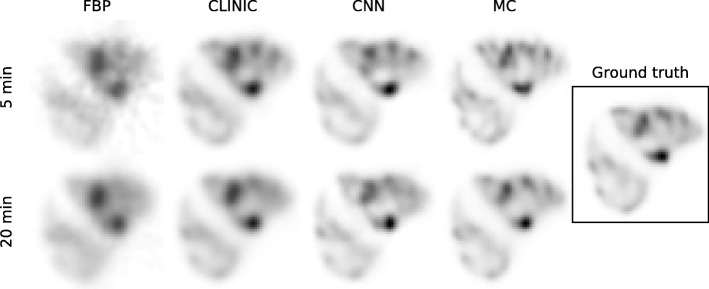
Reconstructed image slices of a representative patient distribution from the validation set, for the four reconstruction methods and two scan times. Additionally shown is the associated ground truth distribution

The reconstructions of one patient distribution from the validation set are shown in Fig. 3 for the four reconstruction methods and two scan times. For all reconstruction methods, it is evident that quality deteriorated with shorter scan time. FBP created reconstructions with severe artefacts and tumour contrast was low. Clinical reconstructions had no visible artefacts but had low contrast too. The CNN approach provided results that were comparable to the Monte Carlo-based reconstructions.

**Fig. 4:**
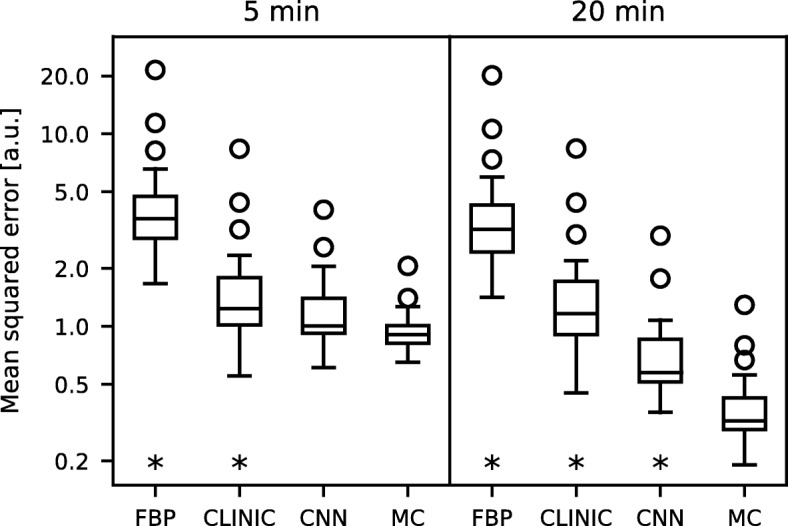
Mean squared error for the 20 distributions in the validation set, for the four reconstructions methods and the two scan times. The asterisk denotes the reconstruction methods that were significantly different from the Monte Carlo-based reconstruction (Mann-Whitney *U* test at *p* < 0.01)

The mean squared error of the reconstructions with the associated ground truth distributions in the validation set is depicted in Fig. 4. These quantitative results showed that the neural network approach had the performance closest to the Monte Carlo-based reconstruction, for both scan times.

**Fig. 5:**
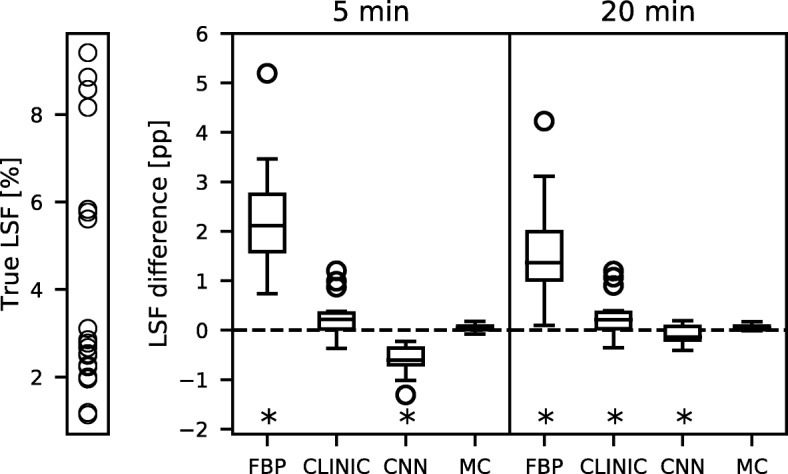
The difference in percent point (pp) of the LSF for the 20 distributions in the validation set, for the four reconstructions methods and the two scan times. Additionally shown are the LSFs found in the ground truth distribution. The asterisk denotes the reconstruction methods that resulted in a significantly different LSF from the Monte Carlo-based reconstruction (Mann-Whitney *U* test at *p* < 0.01)

The difference of the LSF obtained with the four reconstruction methods with that from the ground truth distributions is shown in Fig. 5. The Monte Carlo-based reconstruction and clinical reconstruction provided the lowest difference. FBP resulted in an overestimation of several percent point (pp) and the CNN reconstruction gave a slight underestimation for the 5 min scan. The values from clinical, CNN, and Monte Carlo-based reconstruction were all, however, within 2 pp difference.

### Phantom measurements

The quantitative measures from the reconstructions of the phantom experiment are compiled in Table 1, together with the true values as configured in the phantom. The uptake ratios became relatively worse for spheres of decreasing size, due to the partial volume effect. The Monte Carlo-based and CNN reconstructions provided similar ratios, whereas the found values were lower for clinical reconstruction and FBP. The CNN, clinical, and MC reconstructions gave comparable LSF values; a higher fraction was obtained with FBP. The CNR provided values that were in the same range for both clinical, CNN, and Monte Carlo-based reconstruction; again, only FBP had substantially lower values.

**Table 1:** Lung shunting fraction, uptake ratio for the spheres, and contrast-to-noise ratio for the four reconstruction methods and two scan times, together with the values as configured in the phantom

		LSF [%]	Up. 2.0 mL	Up. 4.1 mL	Up. 8.1 mL	Up. 15.7 mL	CNR
True values		5.2	2.7	2.7	2.7	7.7	–
5 min	FBP	6.4	0.5	0.8	1.0	4.1	6.5
	CLINIC	5.1	1.0	1.4	1.5	5.2	10.3
	CNN	4.7	1.7	2.3	2.0	6.6	12.1
	MC	5.8	2.0	2.3	2.2	6.5	11.0
20 min	FBP	5.3	0.5	0.8	1.0	4.1	6.6
	CLINIC	4.7	1.0	1.4	1.5	5.1	10.5
	CNN	5.1	1.9	2.3	2.3	7.2	12.5
	MC	5.2	2.0	2.2	2.2	6.5	11.6

### Original detector projections

**Fig. 6:**
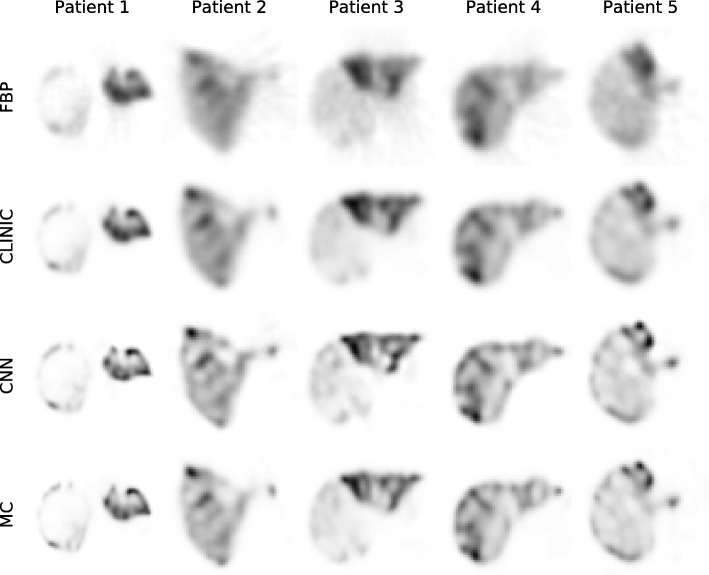
Reconstruction slices of five representative distributions from the test set, for the four reconstruction methods

Five reconstructions obtained from the real detector projections of the 8 patients in the testing set are visualized in Fig. 6. These projection sets were generally obtained a few hours after injection (in contrast to our envisioned interventional scanning) and thus did not carry the 150 MBq as in the simulations. The network from the 20 min scan time was used for the CNN because this network was trained with the closest activity levels. No ground truth is available for these images. Visual inspection, however, showed that CNN and Monte Carlo-based reconstruction had the same features present. This indicated that the neural network approach was also able to generalize well to true detector measurements for patients.

## Discussion

This study showed that FBP with an image enhancement convolutional neural network in SPECT/CT can perform reconstruction within seconds. From the evaluation of the validation set, the phantom experiment, and visual inspection of the testing set, it was found that image quality close to that of the Monte Carlo-based reconstructor was retrieved. The neural network approach additionally performed well for short scans, which would be required for use in interventional SPECT/CT scanning.

This study is one of the first to illustrate the use of deep learning in SPECT. In other modalities, such as positron emission tomography (PET) [24, 25], the use of an image enhancement neural network has previously been explored. The mentioned studies, however, had the primary aim of limiting the injected dose and made no use of the acceleration in reconstruction time as is illustrated in this study. Also, the method of creating synthetic volumes to augment the training data has not shown before in nuclear medicine.

Although this study focused on potential implementation in the pre-treatment procedure of radioembolization, we believe that our approach is applicable to all forms of SPECT/CT. We envision that one use would be to quickly validate whether a scan was made without any motion artefacts. The network in this work was only trained with distributions from radioembolization. It is unknown how well the network generalizes to other distributions. It might prove that the neural network needs to be trained for each procedure separately to ensure optimal results.

In the current clinical practice of radioembolization, the pre-treatment procedure of radioembolization has two major aims: determination of the lung shunting fraction and detection of potential extrahepatic distributions. Both measures were accurately retrieved from the reconstructions from the neural network approach in the phantom experiment. For the patient distributions, it was found that the difference of the LSF obtained with CNN and the ground truth was within 2 percent point, which can be considered clinically sufficient.

A limitation of this study is that it was not shown that extrahepatic depositions were also accurately detected in the patient distributions. Because these depositions are not commonly found in our institute and we did not perform a selection on which distributions to include in training or validation, there were not enough cases to draw conclusions on. However, by means of the phantom experiment and by illustrating the visual quality of the images obtained with CNN, we are confident that patient extrahepatic depositions would also be accurately detected.

It was shown that upon increasing the number of synthetic volumes, the neural network performance increased. A further increase in quality might be achievable when including more volumes or adding more layers to the network. However, this would simultaneously increase the required GPU memory. Such computational aspects are currently holding back further increase in network performance. With a smarter selection of distributions (i.e. assembling the set with the highest diversity), it might be possible to achieve improved results at the same computational cost.

The clinical and Monte Carlo-based reconstructions were performed using a single core in this study. Substantial acceleration can be achieved when the code is parallelized and a dedicated workstation with many CPU cores is used, which might bring the Monte Carlo-based reconstruction time to a clinically acceptable value. The use of GPUs is also very promising for shortening the reconstruction time [26]. An advantage of the neural network approach is that the reconstruction time is almost independent of the matrix size and the number of iterations.

A potential application for the developed method was proposed for interventional scanning in radioembolization. This would also require short scan times and a scanner in the intervention room to ensure a smooth workflow. As for the short scan times, it was recently shown that one can move toward substantially reduced scan times (< 10 min) and still obtain accurate results on various quantitative measures [27]. As for the scanner, a mobile compact system that can be included in the intervention room [7–9] is currently being built and it is expected that this system is available for patient studies soon. Combined with the fast reconstruction in this work, we believe that all elements are present to enable interventional SPECT imaging in clinical practice.

## Conclusions

FBP with an image enhancement convolutional neural network can provide SPECT reconstructions similar in quality to those obtained with Monte Carlo-based reconstruction for the pre-treatment procedure of hepatic radioembolization within seconds.

## Data Availability

All data is stored at the University Medical Center Utrecht, Utrecht, NL.

## Abbreviations

^99m^Tc-MAA: Technetium-99m macroaggregated albumin
CLINIC: Clinical reconstruction
CNN: Convolutional neural network
CNR: Contrast-to-noise ratio
CPU: Central processing unit
FBP: Filtered back projection
GPU: Graphics processing unit
LEHR: Low energy high resolution
LSF: Lung shunting fraction
MC: Monte Carlo-based reconstruction
PET: Positron emission tomography
pp: Percent point
SPECT: Single-photon emission computed tomography
UMCS: Utrecht Monte Carlo System
